# Fatty acids of *Helicobacter pylori* lipoproteins CagT and Lpp20

**DOI:** 10.1128/spectrum.00470-24

**Published:** 2024-03-19

**Authors:** Mark S. McClain, William E. Boeglin, Holly M. Scott Algood, Alan R. Brash

**Affiliations:** 1 Department of Medicine, Vanderbilt University School of Medicine, Nashville, Tennessee, USA; 2 Vanderbilt Institute for Infection Immunology and Inflammation, Vanderbilt University Medical Center, Nashville, Tennessee, USA; 3 Department of Pharmacology, Vanderbilt University School of Medicine, Nashville, Tennessee, USA; 4 Department of Pathology, Microbiology, and Immunology, Vanderbilt University School of Medicine, Nashville, Tennessee, USA; 5 Vanderbilt Center for Immunobiology, Vanderbilt Medical Center, Nashville, Tennessee, USA; 6 Veterans Affairs Tennessee Valley Healthcare System, Nashville, Tennessee, USA; University of Nebraska Medical Center, Omaha, Nebraska, USA

**Keywords:** *Helicobacter pylori*, lipoproteins, phospholipids, acyltransferases, posttranslational protein modification, Toll-like receptor 2

## Abstract

Bacterial lipoproteins are post-translationally modified by the addition of acyl chains that anchor the protein to bacterial membranes. This modification includes two ester-linked and one amide-linked acyl chain on lipoproteins from Gram-negative bacteria. *Helicobacter pylori* lipoproteins have important functions in pathogenesis (including delivering the CagA oncoprotein to mammalian cells) and are recognized by host innate and adaptive immune systems. The number and variety of acyl chains on lipoproteins impact the innate immune response through Toll-like receptor 2. The acyl chains added to lipoproteins are derived from membrane phospholipids. *H. pylori* membrane phospholipids have previously been shown to consist primarily of C14:0 and C19:0 cyclopropane-containing acyl chains. However, the acyl composition of *H. pylori* lipoproteins has not been determined. In this study, we characterized the acyl composition of two representative *H. pylori* lipoproteins, Lpp20 and CagT. Fatty acid methyl esters were prepared from both purified lipoproteins and analyzed by gas chromatography-mass spectrometry. For comparison, we also analyzed *H. pylori* phospholipids. Consistent with previous studies, we observed that the *H. pylori* phospholipids contain primarily C14:0 and C19:0 cyclopropane-containing fatty acids. In contrast, both the ester-linked and amide-linked fatty acids found in *H. pylori* lipoproteins were observed to be almost exclusively C16:0 and C18:0. A discrepancy between the acyl composition of membrane phospholipids and lipoproteins as reported here for *H. pylori* has been previously reported in other bacteria including *Borrelia* and *Brucella*. We discuss possible mechanisms.

**IMPORTANCE:**

Colonization of the stomach by *Helicobacter pylori* is an important risk factor in the development of gastric cancer, the third leading cause of cancer-related death worldwide. *H. pylori* persists in the stomach despite an immune response against the bacteria. Recognition of lipoproteins by TLR2 contributes to the innate immune response to *H. pylori*. However, the role of *H. pylori* lipoproteins in bacterial persistence is poorly understood. As the host response to lipoproteins depends on the acyl chain content, defining the acyl composition of *H. pylori* lipoproteins is an important step in characterizing how lipoproteins contribute to persistence.

## INTRODUCTION

**Fig 1:**
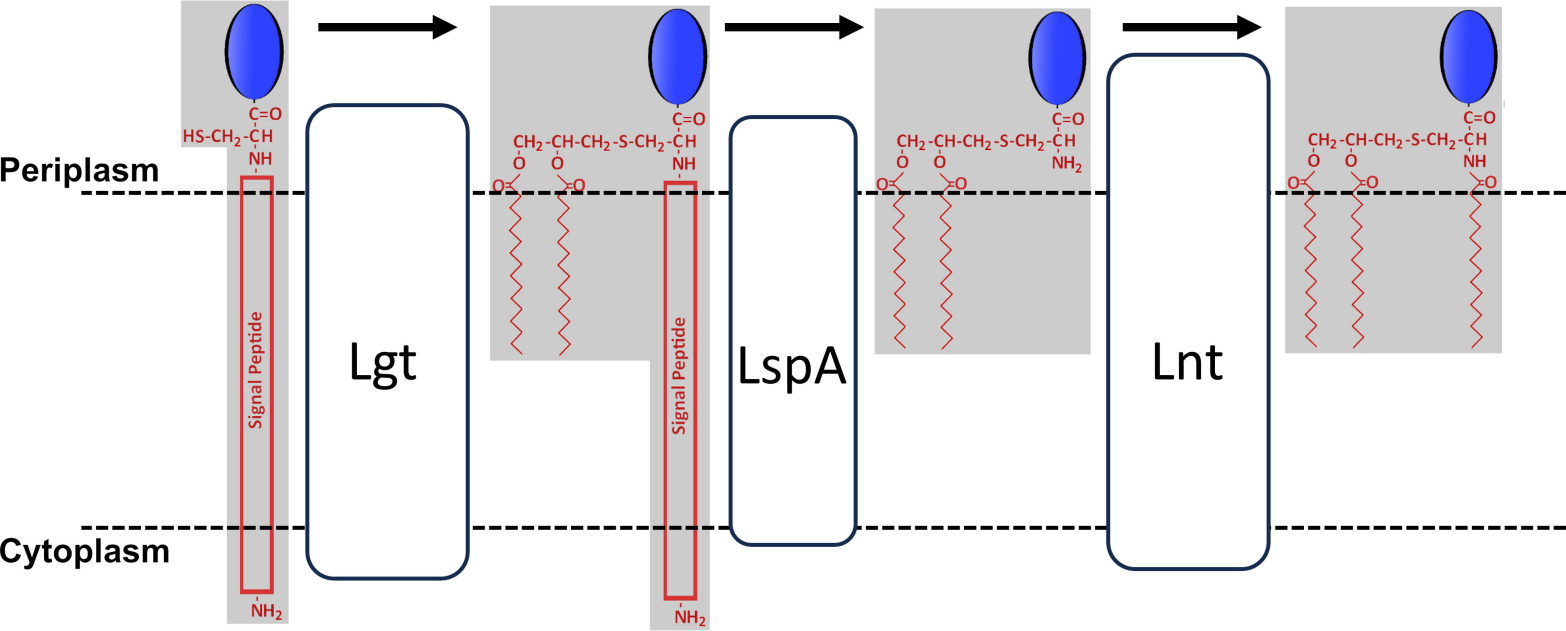
Lipoprotein synthesis. The post-translational modification of lipoproteins is carried out by three membrane-embedded enzymes. First, a short cysteine-containing amino acid sequence known as a lipobox is recognized by prolipoprotein diacylglyceryl transferase (Lgt) which transfers a diacylglyceride with two ester-linked acyl chains from a membrane lipid to the cysteine sulfhydryl of the preprolipoprotein. Second, prolipoprotein signal peptidase (LspA, signal peptidase II) cleaves the amino acids preceding the cysteine, resulting in a diacylated apolipoprotein. Finally, apolipoprotein N-acyltransferase (Lnt) transfers an acyl chain from a membrane lipid to the amino-terminal cysteine via an amide bond to produce the mature triacylated lipoprotein. For simplicity, the lipoprotein is illustrated as uniformly acylated with C16:0.

Bacterial lipoproteins are post-translationally modified by the addition of acyl chains that help anchor the protein to bacterial membranes. Some lipoproteins perform essential functions. For example, the proper assembly of the outer membrane of Gram-negative bacteria requires lipoproteins such as LptE (for assembly of lipopolysaccharide, LPS), BamD (for assembly of outer membrane proteins), and LolB (for proper localization of lipoproteins). In Gram-negative bacteria, lipoproteins are synthesized through a series of conserved enzymatic steps (Fig. 1) (1–3). The acylating enzymes prolipoprotein diacylglyceryl transferase (Lgt) and apolipoprotein N-acyltransferase (Lnt) use membrane phospholipids as acyl chain donors with Lgt transferring a diacylglycerol with two ester-linked acyl chains and Lnt attaching a single acyl chain via an amide linkage (4). The acyl chain composition of lipoproteins might therefore be assumed to reflect the acyl chain composition of membrane lipids. This appears to be true in *Escherichia coli* in which lipoproteins may include C16:0, C16:1, C17:1, C18:1, and C19:1 in approximately the same proportion as found in membrane phospholipids (the possibility that each of the three acylated positions may be modified by a variety of different acyl chains results in significant heterogeneity within a species) (2, 5, 6). However, the acyl chain content of lipoproteins from *Acholeplasma*, *Brucella*, *Borrelia*, and *Treponema* differs markedly from the acyl chain content of the respective membrane phospholipids (7–10).

In addition to their vital roles in bacterial physiology, lipoproteins are a pathogen-associated molecular pattern recognized by mammalian Toll-like receptor 2 (TLR2)-TLR1 or TLR2-TLR6 heterodimers. As the acyl chains of lipoproteins are presumably anchored in the bacterial membrane, lipoproteins likely must be liberated from the bacteria by bacterial or host processes to interact with TLR2 (11, 12). Indeed, studies of extracellular *Helicobacter pylori* proteins report a variety of lipoproteins (13–15). However, variation in the acyl chain composition of lipoproteins can affect TLR2 signaling (16–25). For example, varying the acyl chains of a lipopeptide from two palmitoyl chains (C16:0) to two lauroyl (C12:0), myristoyl (C14:0), or stearoyl (C18:0) chains has been shown to reduce TLR2 activation as determined using TLR2-dependent reporter gene activation assays (25, 26). Variation among acyl chains also may lead to either immune activation or suppression (18, 19). For example, a triacylated lipoprotein modified by a short-chain fatty acid (C2) as the amide-linked acyl chain induced a stronger Th1 cytokine response by dendritic cell/T-cell cocultures compared to cocultures stimulated by the same protein modified by a long chain fatty acid (C17:0) as the amide linked acyl chain (18). Furthermore, bacteria expressing lipoproteins modified with the short-chain fatty acid as the amide-linked acyl chain exhibited elevated TNFα levels and reduced viability in a murine infection model (18). In other studies, dipalmitoylated lipopeptides (lacking the amide-linked acyl chain) reduced TLR2-dependent T-cell-mediated recall response in an atopic dermatitis model when compared to tripalmitoylated lipopeptide (19).

*H. pylori* is a Gram-negative bacterium that persistently colonizes the stomach in about 50% of the human population (i.e., over 4 billion people), eliciting gastric inflammation (27, 28). Without antibiotic therapy, *H. pylori* can persist in the stomach for decades (29). Over time, *H. pylori*-induced gastric inflammation can act as an important initiating event leading some *H. pylori*-infected individuals to develop gastric cancer (27, 30–33). *H. pylori* is predicted to encode about 40 different lipoproteins (34–37). Though many of these lipoproteins have unknown functions, some have been reported to contribute to colonization of the stomach and bacterial adhesion to mammalian cells (38–40), to stimulate IFN-γ production (41), to alter host cell migration and signaling (42, 43), and to contribute to delivery of the CagA oncoprotein to mammalian cells (44–47).

Since TLR2 signaling contributes to immune tolerance toward *H. pylori* infection thereby facilitating long-term bacterial persistence (48, 49) and *H. pylori* lipoproteins contribute to TLR2 activation (41, 50), understanding the acyl composition of lipoproteins is important to our understanding of inflammation and tolerance during this chronically persistent infection. However, the acyl composition of *H. pylori* lipoproteins has not been determined. *H. pylori* has previously been shown to maintain an unusual composition of membrane lipids consisting primarily of phosphatidylethanolamine (PE) carrying a mixture of myristic acid (C14:0) and 19-carbon cyclopropane-containing fatty acids, with lesser quantities of stearic acid (C18:0), *cis*-vaccenic acid (C18:1), and palmitic acid (C16:0) (51–54).

In the current study, we characterize the acyl chain content of two representative *H. pylori* lipoproteins, CagT and Lpp20. We affinity-purified epitope-tagged CagT and Lpp20 from *H. pylori*. CagT is an approximately 30-kDa essential component of the *H. pylori* Cag Type IV secretion system required for the delivery of the CagA oncoprotein to mammalian cells (44–47, 55, 56). We showed in a previous study that the mobility of the N-terminal peptide of CagT in an SDS-PAGE gel differed between wild-type *H. pylori* and a mutant strain in which Lnt had been deleted suggesting that CagT is a lipoprotein (44). Lpp20 is an approximately 17-kDa protein of unknown function reported to stimulate epithelial-mesenchymal transition and chronic immune thrombocytopenia related to *H. pylori* infection (43, 57, 58). Previous proteomic studies suggest that Lpp20 is abundantly expressed in *H. pylori* and can be found both in the membrane (59) and among extracellular *H. pylori* proteins (13–15, 60). We recently showed Lpp20 that is isolated from wild-type vs *lnt* mutant *H. pylori* induces different TLR2-dependent responses consistent with differences between tri- and diacylated control lipopeptides (50). To our knowledge, CagT and Lpp20 are the only two examples of proteins for which evidence has been published indicating that these proteins when isolated from *H. pylori* are indeed lipoproteins.

The lipoprotein acyl transferases Lgt and Lnt are not known to acylate different lipoproteins within the same bacteria with different combinations of acyl chains. On the contrary, evidence indicates that varying the protein sequence does not impact the variety of acyl chains added to lipoproteins (5, 6, 10). Thus, we expect epitope-tagged CagT and Lpp20 to be representative of the *H. pylori* lipoproteome. Additionally, the amino acid sequences of Lgt and Lnt (including amino acid residues corresponding to critical residues identified in the *E. coli* orthologs) are highly conserved among *H. pylori* strains (44, 61–67). Furthermore, studies by multiple groups using multiple *H. pylori* strains and diverse growth conditions find similar acyl chain content among *H. pylori* membrane lipids (51–54, 68). Thus, we believe that analyzing the acyl chain content of representative lipoproteins from strain 26695 is representative of the species.

We liberated the fatty acids from the purified proteins and identified the fatty acid methyl esters by gas chromatography-mass spectrometry (GC-MS). The acyl chain composition of these *H. pylori* lipoproteins was compared to the fatty acids liberated from purified *H. pylori* membrane lipids. Results showed that the *H. pylori* lipoproteins are modified with palmitic and stearic acids which are minor components of the *H. pylori* membrane lipids. These results suggest pronounced lipid donor specificity of the *H. pylori* Lgt and Lnt enzymes and may impact the host response to *H. pylori* lipoproteins.

## RESULTS

### Fatty acid composition of *H. pylori* cellular lipids

**Fig 2:**
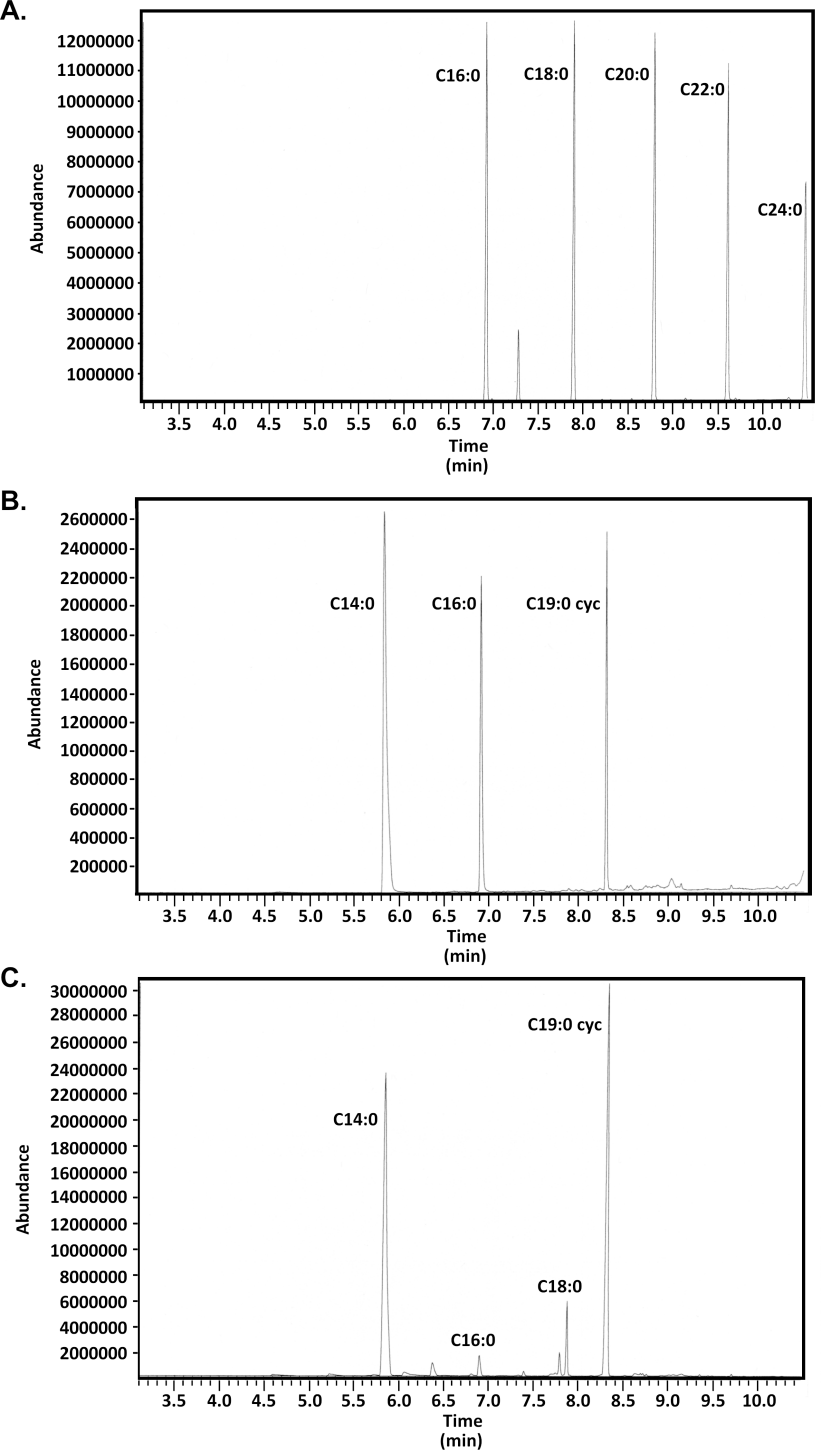
Gas chromatograms of fatty-acid methyl esters (FAMEs). (A and B) Commercially available FAMEs were combined to form two standard preparations containing palmitic, stearic, arachidonic, behenic, and lignoceric acid methyl esters (**A**), or myristic, palmitic, and *cis*-9,10-methyleneoctadecanoic acid methyl esters (**B**). (**C) ***H. pylori* phospholipids were extracted and converted to FAMEs as described in Materials and Methods. The resulting FAMEs were analyzed by GC-MS. The results are representative of four or more independent samples. The chemical identities were determined based on elution relative to standards and confirmed based on the fragmentation patterns (data not shown).

Because the acyl chains added to bacterial lipoproteins are derived from membrane lipids, we first sought to confirm the acyl composition of *H. pylori* phospholipids. We isolated lipids from *H. pylori* strain 26695. Strains were grown in *Brucella* broth cultures, and lipids were isolated using a modified Folch procedure (69) as described in Materials and Methods. Though *H. pylori* is often considered to be highly diverse, previous studies have analyzed a variety of different *H. pylori* strains that were grown under a wide variety of different conditions and reached similar conclusions regarding the acyl composition of *H. pylori* membrane phospholipids (52–54, 68). Results of these studies suggest that the acyl composition of *H. pylori* phospholipids is a fundamental attribute of *H. pylori*. Fatty acids may be released via acid or alkaline hydrolysis, though acid hydrolysis is more effective than alkaline hydrolysis at hydrolyzing amide-linked fatty acids (70). However, acid hydrolysis also leads to ring opening and generation of methoxyester derivatives of cyclopropane-containing acyl chains (71–73). To avoid this in the present study, fatty acids were released from the lipids and converted to fatty acid methyl esters through sequential alkaline saponification, acid hydrolysis, and esterification which has been shown to preserve cyclopropane-containing fatty acids (72). The fatty acid methyl esters were analyzed by GC-MS and compared to analytical standards (Fig. 2). Analyses of the gas chromatograms and fragmentation patterns revealed that *H. pylori* lipids contain primarily myristic acid and cyclopropane-containing fatty acids (Fig. 2C). These results are consistent with those of the previous studies (52–54, 68).

### Analysis of *H. pylori* lipoprotein-derived FAMEs

To determine the fatty acid content of representative *H. pylori* lipoproteins, derivatives of *H. pylori* strain 26695 expressing a DDK-tagged version of CagT (strain BV357) (44) or a human influenza hemagglutinin (HA)-tagged version of Lpp20 (strain VM396) (50) were grown in broth culture under the same conditions used when isolating *H. pylori* lipids. CagT-DDK and Lpp20-HA were purified by immunoprecipitation as described in Materials and Methods. Fatty acids were released from the purified lipoproteins and converted to fatty acid methyl esters through the sequential alkaline saponification, acid hydrolysis, and esterification methods (72). In addition to preserving cyclopropane-containing fatty acids, this method has been shown to liberate both ester- and amide-linked fatty acids (72).

**Fig 3:**
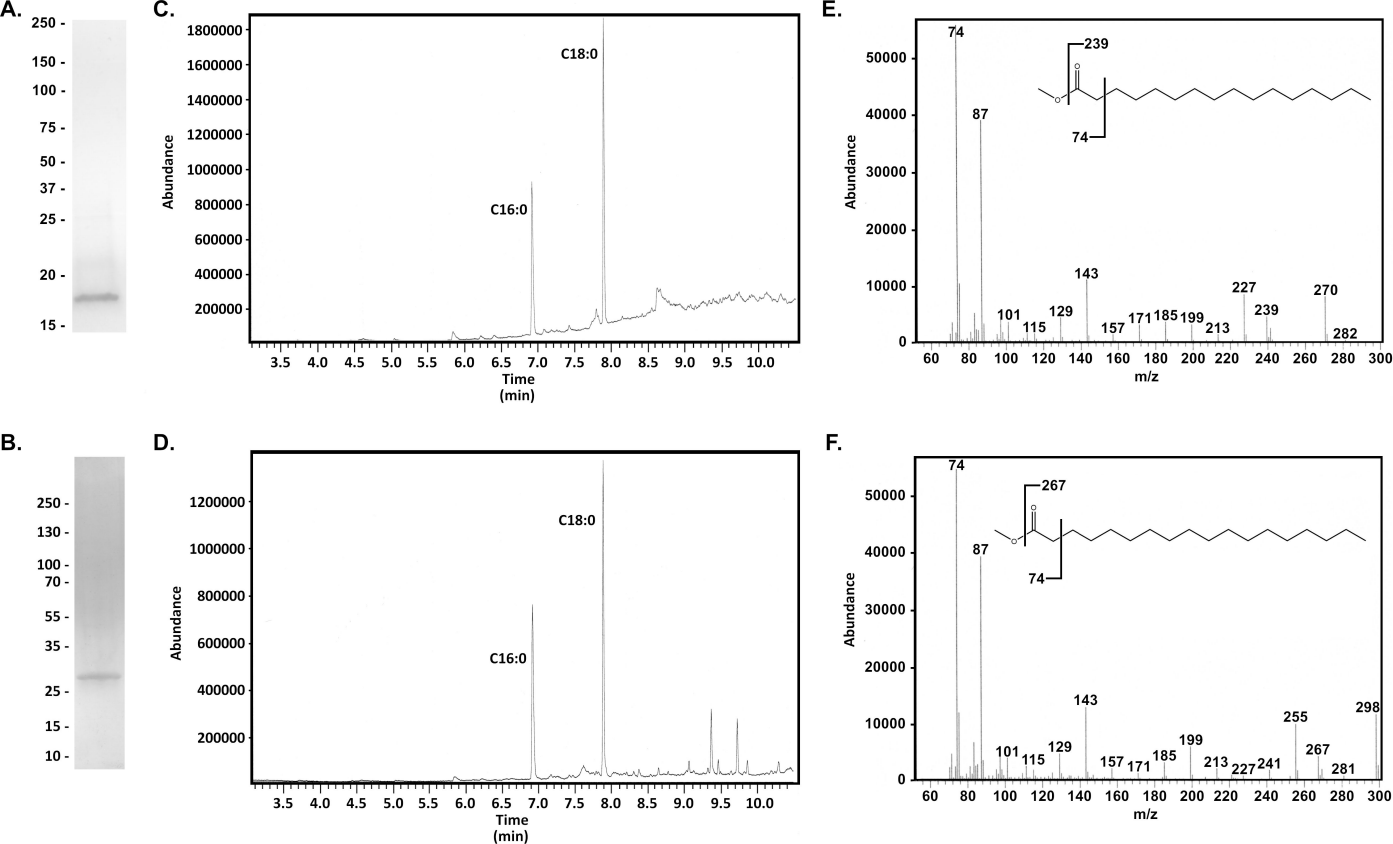
GC-MS analysis of fatty-acid methyl esters derived from *H. pylori* lipoproteins. (A and B) Coomassie-stained gels of purified Lpp20-HA (**A**) and CagT-DDK (**B**). (C and D) The acyl chains were hydrolyzed from purified Lpp20-HA and CagT-DDK and converted to FAMEs as described in Materials and Methods. The resulting FAMEs were analyzed by GC-MS (**C**, Lpp20-HA; **D**, CagT-DDK). The gas chromatograms are representative of three or more independent samples of each lipoprotein. The elution times for the 6.9- and 7.9-minute peaks are consistent with the elution times of palmitic acid methyl ester and stearic acid methyl ester standards, respectively (Fig. 2). Analysis of the MS fragmentation patterns of molecules eluting at 9.4 and 9.7 minutes suggests that these are contaminants (e.g., from labware) rather than CagT-DDK-derived fatty acid methyl esters. (E and F) MS fragmentation patterns of the 6.9-minute peak (**E**) and 7.9-minute peak (**F**) are consistent with the expected fragmentation patterns of palmitic acid methyl ester (**E**) and stearic acid methyl ester (**F**). Insets illustrate characteristic fragment ions seen in FAMES. The series of ions uniformly 14 amu apart (e.g., at *m*/*z* = 87, 101, 115, 129, 143, 157, 199, and so forth) is evidence that there are unlikely to be other functional groups in the acyl chain.

The fatty acid methyl esters were analyzed by GC-MS and compared to analytical standards (Fig. 3). Analyses of the gas chromatograms and MS fragmentation patterns revealed that *H. pylori* lipoproteins contain primarily palmitic and stearic acid with little if any myristic acid or C19:0 cyclopropane-containing fatty acids (Fig. 3). These results are in stark contrast to the results observed for *H. pylori* membrane lipids (Fig. 2). The analyses of lipoprotein also contain little if any 3-hydroxy palmitic or 3-hydroxy stearic acids characteristic of *H. pylori* LPS (as evidenced in part by the lack of characteristic ions with *m*/*z* of 103 in the MS analysis) (52).

### Positional distribution of acyl chains in *H. pylori* lipoproteins

**Fig 4:**
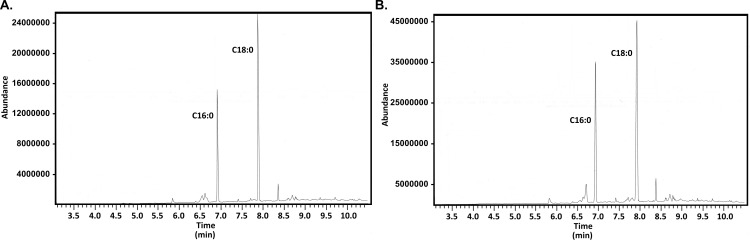
Gas chromatograms of ester- and amide-linked fatty-acid methyl esters. (A) The ester-linked acyl chains of CagT-DDK were hydrolyzed under alkaline conditions and converted to FAMES for analysis by GC-MS. The chemical identities were determined based on elution relative to standards and confirmed based on the fragmentation patterns (data not shown). Results reveal a mixture of palmitic and stearic acids. Similar results were obtained when analyzing acyl chains released from Lpp20-HA by lipoprotein lipase and when analyzing CagT-DDK and Lpp20-HA isolated from *lnt* mutant *H. pylori* (which contain the ester-linked acyl chains but not the amide-linked acyl chain, data not shown). (B) CagT-DDK remaining following alkaline hydrolysis (panel A) was treated under acidic conditions to hydrolyze the amide-linked acyl chain which was subsequently converted to FAMES and analyzed by GC-MS. Results reveal a mixture of palmitic and stearic acids. Similar results were obtained when analyzing CagT-DDK which had first been treated with lipoprotein lipase to remove ester-linked acyl chains and then treated under acidic conditions to hydrolyze the amide-linked acyl chain. Similar results also were obtained by treating Lpp20-HA with lipoprotein lipase to remove the ester-linked fatty acids, followed by acid hydrolysis of the remaining amide-linked fatty acid (data not shown).

To identify acyl chains added by Lgt, we next sought to characterize the two ester-linked fatty acids of *H. pylori* lipoproteins. As one approach, we isolated CagT-DDK from *H. pylori* strain BV357. Alkaline hydrolysis was used to hydrolyze the ester-linked fatty acids while sparing the amide-linked fatty acid, and methyl esters were generated and analyzed by GC-MS (70). Analyses of the gas chromatograms and MS fragmentation patterns revealed a mixture of primarily palmitic and stearic acids (Fig. 4A).

To identify the acyl chain added by Lnt, we sought to characterize the amide-linked fatty acid of *H. pylori* lipoproteins. As one approach, we isolated CagT-DDK from *H. pylori* strain BV357. The ester-linked fatty acids were removed by alkaline hydrolysis, and the sample was extracted with chloroform to remove the liberated fatty acids. The residual protein containing the amide-linked fatty acid was then subjected to acid hydrolysis to remove the amide-linked fatty acid. Methyl esters were then generated and analyzed by GC-MS. Analyses of the gas chromatograms and MS fragmentation patterns revealed a mixture of primarily palmitic and stearic acids (Fig. 4B). Results suggest that the amide-linked acyl chain may be either C16:0 or C18:0 (though we cannot rule out the possibility that these results might also contain residual ester-linked acyl chains).

## DISCUSSION

Two membrane-embedded enzymes Lgt and Lnt transfer a diacylglyceride or a single fatty acid chain, respectively, from membrane lipids to newly synthesized lipoproteins. In their pioneering work in the field, Hantke and Braun (5) characterized similar fatty acid compositions in *E. coli* phospholipids and in Braun’s lipoprotein (Lpp). This has been confirmed in subsequent studies examining different lipoproteins expressed in *E. coli* (6). *H. pylori* membrane lipid composition is recognized for consisting primarily of myristic acid and 19-carbon cyclopropane-containing fatty acids (51–54). This study characterizes for the first time the acyl chain composition of lipoproteins isolated from *H. pylori*.

In the present study, we hydrolyzed both ester-linked and amide-linked fatty acids from two lipoproteins, CagT and Lpp20. The free fatty acids were converted to methyl esters, and the fatty acid methyl esters were identified by GC-MS. Results demonstrated palmitic (C16:0) and stearic (C18:0) fatty acids. Our selective analyses of ester-linked acyl chains (added by Lgt) and the amide-linked acyl chain (added by Lnt) of representative *H. pylori* lipoproteins suggest that both acylating enzymes use palmitic and stearic acid-containing lipids as acyl chain donors. The acyl chain content of *H. pylori* lipoproteins thus stands in marked contrast to the composition of *H. pylori* membrane lipids which primarily consist of C14:0 and C19 cyclopropane-containing fatty acids (51–54). Similarly, the acyl chain composition of lipoproteins has been reported to diverge from the acyl chain composition of membrane lipids in *Acholeplasma*, *Brucella*, *Borrelia*, and *Treponema* (7–10).

Differences between the acyl composition of membrane lipids and lipoproteins as reported here and in previous studies may reflect the substrate preferences of the acylating enzymes Lgt and Lnt (7–10). Analyses of the *E. coli* orthologs indicate specificity for the phospholipid headgroup with little specificity for acyl chain length (61). For example, *E. coli* Lgt preferentially uses phosphatidylglycerol (PG) phospholipids with various acyl chain lengths, whereas *E. coli* Lnt preferentially uses PE phospholipids with a preference for acyl chains longer than C12 (61, 67, 74). *H. pylori* phospholipids consist primarily of PE and PG. If headgroup specificity by *H. pylori* Lgt or Lnt was to account for the observed discrepancy of acyl chain content between lipoproteins and the phospholipid membrane, one would expect that *H. pylori* PE or PG would be modified predominantly by C16:0 and C18:0. However, analysis of the acyl chain composition of *H. pylori* PE and PG reveals that both phospholipids contain primarily C14:0 and cyclopropane-containing C19:0, similar to the total phospholipid (51). Thus, headgroup specificity likely cannot account for the acyl chain selectivity of *H. pylori* Lgt and Lnt. Alternatively, discrepancies between the acyl chain composition of lipoproteins and membrane phospholipids could reflect a preferred lipid environment that stabilizes the activity of the acylating enzymes. For example, dipalmitoyl PG was found to be more effective than 1-palmitoyl-2-oleoyl-PG at enhancing the thermostability of *E. coli* Lgt (61). Although cholesterol-rich membrane microdomains have been suggested in *H. pylori* (75), we are not aware of evidence or theoretical models in which PE or PG containing C16:0 and/or C18:0 acyl chains form membrane microdomains within membranes comprised primarily of PE or PG containing C14:0 and cyclopropane-containing C19:0 acyl chains. A third possible explanation is based on selectivity from within the enzyme active sites. Although it is not clear that *E. coli* Lgt and Lnt exhibit strong preferences for acyl chain lengths, the acyl chain-binding pockets in a variety of other bacterial acyltransferases act as highly selective “molecular rulers” (76–83). For example, LpxD is specific for 14-carbon acyl chains in *E. coli* and for 20-carbon acyl chains in *Chlamydia trachomatis* (82, 83 ), and PagP can distinguish acyl chains that differ by a single methylene unit (76). In addition to selecting certain acyl chain lengths, the acyl chain-binding pockets of *H. pylori* Lgt and Lnt may exclude cyclopropane-containing acyl chains due to the rigid bend introduced by the cyclopropane ring (84). We suggest that acyl chain selectivity by *H. pylori* Lgt and Lnt is due to molecular rulers.

The contributions of *H. pylori* lipoproteins to the mammalian inflammatory response are not fully understood. A previous study showed that recombinant *H. pylori* HpaA (purified from *E. coli* and therefore with an acyl chain composition like that of *E. coli* lipoproteins) was able to stimulate IFNγ from human natural killer cells (41). We recently showed that Lpp20 isolated from *H. pylori* induces transcription of numerous chemokines and cytokines from primary human gastric epithelial cells (50). However, we also showed that the response to Lpp20 from wild-type *H. pylori* was less robust than the response to the synthetic tripalmitoylated peptide Pam3CSK4 and that the response to Lpp20 from an *lnt* mutant *H. pylori* strain was less robust than the response to the synthetic dipalmitoylated peptide Pam2CSK4. Given that stearoyl lipopeptides have been reported to be less active than palmitoyl lipopeptides (25, 26) and results of the present study demonstrating that *H. pylori* lipoproteins may contain various mixtures of palmitic and stearic acid at the three acylated positions, further studies are needed to more fully explore the interaction between *H. pylori* lipoproteins and the host innate immune response.

## MATERIALS AND METHODS

### Bacterial strains and culture conditions

*H. pylori* strains (Table 1) were grown on either trypticase soy agar plates containing 5% sheep blood or in bisulfite-free *Brucella* broth supplemented with 1× Cholesterol Lipid Concentrate (Life Technologies), at 37°C in room air supplemented with 5% CO_2_. *H. pylori* mutant strains were selected using kanamycin (12.5 µg mL^−1^) or chloramphenicol (2.5 µg mL^−1^).

**TABLE 1:** Bacterial strains

Strain	Genotype	References
26695		(85)
BV357	26695 Δ*rdxA* Δ*cagT ureA*:(*cagT*-DDK_26,_*cat*) (*Mtz^r^*, Chl^r^)	(44)
VM207	BV357 Δ*lnt::aphA3* (Mtz^r^, Kan^r^, Chl^r^)	(44)
VM396	26695 Δ*rdxA* Δ*cagT ureA*::(*lpp20*-HA, *cat*) (*Mtz^r^*, Chl^r^)	(50)
VM404	VM396 Δ*lnt::aphA3* (Mtz^r^, Kan^r^ Chl^r^)	(50)

### *H. pylori* membrane lipid isolation

*H. pylori* lipids were isolated using a modified Folch method (69). Strains were grown to late log phase in 10-mL bisulfite-free *Brucella* broth supplemented with cholesterol. Cultures were pelleted for 10 minutes at 7500 × *g* in glass tubes. Bacterial pellets were washed in 150-mM NaCl. The washed pellets were then resuspended in 0.3-mL chloroform. Methanol (0.6 mL) was added, and samples were homogenized. To the homogenized samples, 0.3-mL chloroform followed by 0.3 mL of endotoxin-free water were added, and the samples were homogenized. After phase separation, a glass pipet was used to transfer the lower organic phase to a new glass tube. This organic layer was extracted twice with endotoxin-free water. The final organic phase was evaporated under nitrogen to dryness and resuspended in hexane.

### Purification of Lpp20-HA and CagT-DDK

*H. pylori* strains BV357, VM207, VM394, and VM404 (Table 1) were grown in bisulfite-free *Brucella* broth supplemented with cholesterol. Bacteria were pelleted (5,000 × *g*, 10 minutes), and the pellets were resuspended in 1-M 3-(N-morpholino)propanesulfonic acid (MOPS), pH 7 containing protease inhibitors (complete protease inhibitor), and 1,000 units mutanolysin (Sigma) per gram of bacterial pellet. The bacterial suspensions were incubated with rocking at room temperature for 1 hr then passed through a French Press (Glen Mills) at 18,000 psi. Unlysed bacteria were pelleted (7,500 × *g*, 10 minutes) and bacterial membranes were pelleted (174,000 × *g*, 2 hr, 4°C). The total membrane pellet was solubilized in 50-mM MOPS, pH 7, 300-mM NaCl, and 1% Fos-choline 12. Solubilized membranes were incubated with anti-DYKDDDDK or anti-HA-coated magnetic agarose beads (Pierce), and the samples were mixed gently at room temperature for 1 hr. The beads were collected on a magnet and washed four times in 50-mM MOPS, pH 7, 300-mM NaCl, and 1% Fos-choline 12. Affinity-purified proteins were eluted by incubating the beads twice with 50 µL of 2.5 mg mL^−1^ DDK or HA peptide in 50-mM MOPS, pH 7, 300-mM NaCl, and 1% Fos-choline 12. Eluted proteins were buffer exchanged into 50-mM MOPS, pH 7, 300-mM NaCl using Amicon Ultra ultrafiltration devices (3,000-mw cutoff, Millipore), precipitated with acetone, and dried.

### Production of fatty acid methyl esters

Fatty acid methyl esters were generated essentially as described (72). The samples (total *H. pylori* lipids and purified lipoproteins) were suspended in 0.1-mL 1-M KOH, transferred using a glass pipet to hydrolysis tubes (Pierce), and heated to 55°C in a heating block filled with mineral oil for 60 minutes. Freshly diluted HCl (0.3 mL of 2.67 M in endotoxin-free water) was added to the samples in hydrolysis tubes followed by 0.1-mL n-heptane. The samples were then incubated at 80°C overnight in a heating block filled with mineral oil. The samples were cooled to room temperature and extracted with 0.5-mL chloroform. The aqueous phase was discarded. HCl (0.01 mL of concentrated acid) and 0.25-mL methanol were added to each sample. The samples were incubated at 55°C for 30 minutes. The samples were then extracted with 2.5-mL saturated NaHCO_3_ followed by three washes with endotoxin-free water. The samples were then evaporated to dryness under nitrogen and resuspended in hexane.

For the selective analysis of ester-linked acyl chains, lipoprotein was suspended in 0.1-mL 1-M KOH, transferred using a glass pipet to hydrolysis tubes (Pierce), and heated to 55°C in a heating block filled with mineral oil for 60 minutes. The sample was extracted with 0.1-mL hexane followed by extraction with 0.5-mL chloroform. The aqueous phase was saved for analysis of the amide-linked acyl chain. The hexane and chloroform extractions were pooled. HCl (0.01 mL of concentrated acid) and 0.25-mL methanol were added, and the samples were incubated at 55°C for 30 minutes. The samples were then extracted with 2.5-mL saturated NaHCO_3_ followed by three washes with endotoxin-free water. The samples were then evaporated to dryness under nitrogen and resuspended in hexane.

For the selective analysis of the amide-linked acyl chain, freshly diluted HCl (0.3 mL of 2.67 M in endotoxin-free water) was added to the aqueous layer described above. The samples were overlayed with 0.1-mL n-heptane and incubated at 80°C overnight in a heating block filled with mineral oil. The samples were then cooled to room temperature and extracted with 0.5-mL chloroform. The aqueous phase was discarded. HCl (0.01 mL of concentrated acid) and 0.25-mL methanol were added to each sample. The samples were incubated at 55°C for 30 minutes. The samples were then extracted with 2.5-mL saturated NaHCO_3_ followed by three washes with endotoxin-free water. The samples were then evaporated to dryness under nitrogen and resuspended in hexane.

### GC-MS analysis

Fatty acid methyl ester standards were purchased from Cayman Chemical (Ann Arbor, MI, USA). Analysis of fatty acid methyl esters was performed using an Agilent/J and W DB-5MS column (30 m × 0.250 mm, 0.25-µm film) in an Agilent 6890 GC/5973 MSD instrument operated in the electron ionization mode (ion source temperature 230°C, electron energy 70 eV) set to full scans from *m*/*z* 70 to 600. The samples were injected at 130°C, and after 1 minute, the temperature was programmed to 300°C at 20°C min^−1^.
